# Editorial: Tailoring treatment in invasive and non-invasive cervical pathology volume II

**DOI:** 10.3389/fsurg.2024.1540134

**Published:** 2025-01-07

**Authors:** Violante Di Donato, Tullio Golia D’Augè, Alejandro Soderini, Andrea Giannini, Francesco Plotti

**Affiliations:** ^1^ Department of Maternal and Child Health and Urological Sciences, Sapienza University of Rome, Policlinico Umberto I, Rome, Italy; ^2^ Unit of Gynecologic Oncology, Marie Curie Oncologic Hospital, University of Buenos Aires, Buenos Aires, Argentina; ^3^ Unit of Gynecology, Department of Surgical and Medical Sciences and Translational Medicine, Sant’Andrea Hospital, Sapienza University of Rome, Rome, Italy; ^4^ Research Unit of Gynaecology, Department of Medicine and Surgery, Università Campus Bio-Medico di Roma, Rome, Italy

**Keywords:** cervical cancer management, immunotherapy, cost-effectiveness, diagnostic advancements, gynecological pathology

**Editorial on the Research Topic**
Tailoring treatment in invasive and non-invasive cervical pathology volume II

Cervical cancer remains a major global health concern, ranking as the fourth most common cancer among women, with approximately 660,000 new cases and 350,000 deaths recorded in 2022 (1). This disease disproportionately affects women in low- and middle-income countries, where limited access to HPV vaccination, cervical screening, and treatment services reveals deep-rooted health inequities linked to socio-economic determinants (2, 3). Prevention strategies, including HPV vaccination and screening for pre-cancerous lesions, have proven highly effective and cost-efficient (4, 5). Early diagnosis and timely treatment can cure cervical cancer, and countries worldwide are aiming to eliminate this disease by 2030 through targeted global initiatives.

The field of cervical cancer treatment and diagnosis is undergoing a dynamic transformation, cleared by novel approaches that aim to optimize equally therapeutic efficacy and cost-effectiveness (3, 6). The main aim of this Research Topic was to focus on advancing treatment approaches, diagnostic accuracy, and cost-effectiveness in cervical cancer management, addressing both therapeutic efficacy and quality-of-life outcomes for patients with recurrent, persistent, or advanced disease. Recent studies within this research collection highlight pivotal developments, addressing the challenges of this cancer and proposing modern diagnostic procedures. Five high-quality papers were published on this Research Topic: three original research, one clinical trial and one case report.

Together, these studies underscore the complexity of managing cervical cancer, balancing the efficacy of treatments with their financial implications, and the evolving strategies to improve patient outcomes.

The first study, by Lin et al. focuses on the economic analysis of integrating pembrolizumab with chemotherapy and bevacizumab in treating advanced cervical cancer. The partitioned survival model used in this research, based on data from the KEYNOTE-826 trial, reveals an additional 1.18 quality-adjusted life years (QALYs) with this combination, although at a high incremental cost-effectiveness ratio (ICER). Interestingly, cost-effectiveness was notably improved in patients with a programmed death-ligand 1 combined positive score (PD-L1 CPS) ≥ 10, suggesting that personalized adjustments, such as pembrolizumab price adaptation, may make this regimen feasible in select populations. This research emphasizes the importance of tailoring high-cost therapies in line with specific biomarker profiles to enhance economic feasibility, especially in healthcare systems where cost considerations significantly impact treatment accessibility.

Regarding immunotherapy, the second study published by Choi et al. assesses BVAC-C, an immunotherapeutic vaccine, for recurrent cervical carcinoma associated with HPV 16–18. This phase IIa study demonstrates the vaccine's antitumoral effect and practicable safety profile, offering a promising second-line option for patients who have relapsed post-chemotherapy. Notably, BVAC-C induced significant E6/E7-specific T-cell responses and inflammatory cytokine responses correlated with clinical outcomes, indicating the potential of this vaccine to provide long-lasting responses in patients with limited therapeutic options. However, further research is warranted to identify predictive biomarkers of response, which could enhance the clinical utility of BVAC-C in immunotherapy-resistant cervical cancer subtypes.

A case report by Hu et al. presents a rare but important clinical challenge of acquired vulvar lymphangioma (AVL) secondary to cervical cancer surgery, highlighting the requirement for awareness of this potential postoperative complication. This patient, treated with staged surgical excisions, exhibited substantial improvement without recurrence after a year, suggesting that a modulated resection approach may offer a sustainable protocol for managing extensive AVL. This case study serves as a cautionary reminder to clinicians to monitor for AVL in patients with histories of cervical surgery and focuses on the importance of individualized surgical planning for complex cases.

The study by Suchońska et al. turns focus to the management of high-grade squamous intraepithelial lesions (HSIL) following incomplete conization. This study shows that close monitoring, including HPV typing, is crucial for patients of reproductive age to reduce the risks of persistence or recurrence without the necessity for radical surgical interventions. HPV infection was identified as a major risk factor, increasing the chance of recurrent lesions by 38 times. These findings support an approach that balances oncologic control with fertility preservation, emphasizing HPV typing and vaccination as proactive measures to moderate invasive procedures. Lastly, the study by Cui et al. explores the diagnostic potential of optical coherence tomography (OCT) in triaging high-risk HPV-positive patients for early detection of cervical intraepithelial neoplasia (CIN) 2 + and CIN3 + lesions. With higher specificity compared to traditional cytology and an impressive area under the curve (AUC) when combined with HPV genotyping, OCT was demonstrated to be promising in the early detection of cervical abnormalities. This method could significantly refine the triage process, enabling targeted biopsies and reducing unnecessary procedures. The study's findings suggest that OCT may soon play a pivotal role in precision diagnostics for cervical cancer screening programs, particularly for high-risk populations.

In summary, these contributions illuminate the current landscape of cervical cancer management, from innovative immunotherapies and personalized treatment cost-analysis to advanced diagnostic tools and nuanced approaches to surgical management. Together, they reflect a shift towards personalized, biomarker-driven strategies that prioritize efficacy, economic feasibility, and patient quality of life. Future research should continue to investigate predictive biomarkers and refine diagnostic algorithms, striving toward a more individualized, efficient, and accessible approach to cervical cancer care.
